# Processing and Ex Vivo Expansion of Adipose Tissue-Derived Mesenchymal Stem/Stromal Cells for the Development of an Advanced Therapy Medicinal Product for use in Humans

**DOI:** 10.3390/cells10081908

**Published:** 2021-07-27

**Authors:** Anna Labedz-Maslowska, Agnieszka Szkaradek, Tomasz Mierzwinski, Zbigniew Madeja, Ewa Zuba-Surma

**Affiliations:** 1 Department of Cell Biology, Faculty of Biochemistry, Biophysics and Biotechnology, Jagiellonian University, 30-387 Krakow, Poland; anna.labedz-maslowska@uj.edu.pl (A.L.-M.); agnieszka.szkaradek@doctoral.uj.edu.pl (A.S.); z.madeja@uj.edu.pl (Z.M.); 2 Cell & Tissue Culture Laboratory, Jagiellonian Center of Innovation in Krakow, 30-384 Krakow, Poland; mierzw@gmail.com

**Keywords:** adipose tissue-derived mesenchymal stem/stromal cells, advanced therapy medicinal product, cell-based therapy, good manufacturing practices, regenerative medicine

## Abstract

Adipose tissue (AT) represents a commonly used source of mesenchymal stem/stromal cells (MSCs) whose proregenerative potential has been widely investigated in multiple clinical trials worldwide. However, the standardization of the manufacturing process of MSC-based cell therapy medicinal products in compliance with the requirements of the local authorities is obligatory and will allow us to obtain the necessary permits for product administration according to its intended use. Within the research phase (RD), we optimized the protocols used for the processing and ex vivo expansion of AT-derived MSCs (AT-MSCs) for the development of an Advanced Therapy Medicinal Product (ATMP) for use in humans. Critical process parameters (including, e.g., the concentration of enzyme used for AT digestion, cell culture conditions) were identified and examined to ensure the high quality of the final product containing AT-MSCs. We confirmed the identity of isolated AT-MSCs as MSCs and their trilineage differentiation potential according to the International Society for Cellular Therapy (ISCT) recommendations. Based on the conducted experiments, in-process quality control (QC) parameters and acceptance criteria were defined for the manufacturing of hospital exemption ATMP (HE-ATMP). Finally, we conducted a validation of the manufacturing process in a GMP facility. In the current study, we presented a process approach leading to the optimization of processing and the ex vivo expansion of AT-MSCs for the development of ATMP for use in humans.

## 1. Introduction

Mesenchymal stem/stromal cells (MSCs) represent one of the most investigated type of adult stem cells (SCs). MSCs can be isolated from many tissues, including bone marrow (BM), adipose tissue (AT), mobilized peripheral blood, umbilical cord blood, umbilical cord Wharton’s jelly, and dental pulp [1,2]. In 2006, the International Society for Cellular Therapy (ISCT) proposed minimal criteria for defining MSCs of various origins, including (1) an ability to adhere to plastic surfaces under standard culture conditions in vitro; (2) a specific antigenic profile—the presence of CD73, CD90, and CD105 antigens (at least 95% of positive cells) and a parallel lack of the following antigens: CD45, CD34, CD14 or CD11b, CD79α or CD19, and HLA-DR (less than 2% of positive cells); and (3) the capacity to differentiate into mesodermal lineages, including osteoblasts, chondroblasts, and adipocytes in vitro [3].

A number of different mechanisms of MSC action may be responsible for their pro-regenerative activity in injured tissues, including their (1) direct differentiation capacity, leading to direct cell replacement in a damaged tissue, and (2) paracrine activity relying on the secretory capacity of MSCs, including the release of multiple soluble bioactive molecules as well as extracellular vesicles (EVs), which, all together, may indirectly stimulate endogenous processes of tissue repair [4]. MSCs of various origins predominantly exhibit an ability to differentiate into various types of mesodermal cells, such as chondroblasts/chondrocytes, osteoblasts, and adipocytes [5]. It has been shown that BM-derived MSCs (BM-MSCs) differentiating into chondrocytes also secrete proteoglycans and collagen II, which are essential components of the extracellular matrix in cartilage tissue [6]. Importantly, it has been also demonstrated that, upon intra-articular administration into injured joint, BM-MSCs can induce cartilage replacement and be detected in the newly formed tissue [7]. 

The paracrine factors released by MSCs may regulate several processes favoring tissue repair, including angiogenesis and cell proliferation, migration, and survival; they may also be able to reduce inflammation in damaged tissue [4]. The immunosuppressive properties of native MSCs rely on the secretion of a number of anti-inflammatory molecules, including, e.g., interleukin (IL) 6 and IL-10, transforming growth factor β (TGF-β), and prostaglandin E2 (PGE2) [8,9,10]. Thus, native MSCs, due to their immunomodulatory properties, may inhibit the maturation and functions of major classes of immunocompetent cells, such as monocytes, granulocytes, lymphocytes B and T (CD8), and natural killer cells, leading to a decrease in immune reaction [8,9,10]. However, it has been demonstrated that MSCs are capable of sensing and responding to cytokines and factors present in a specific microenvironment, including pro-inflammatory cytokines such as IL-1, IL-17, and interferon γ (IFN-γ) [11,12,13]. Interestingly, MSCs treated and activated with IFN-γ may express a higher level of HLA-DR molecules [14] or HLA-I antigens [15]. Surprisingly, it has been shown that such IFN-γ-activated MSCs did not stimulate allo-reactive T cells but, rather, enhanced their immunosuppressive capacity [13,14], suggesting that MSCs primed with IFN-γ may be used for the treatment of allogeneic conflicts in patients, including graft-versus-host disease [13]. Considering the phenomenon of cytokine sensing in the MSC microenvironment, which may impact on the immunomodulatory properties of these cells, an optimal process of propagation is required prior to their clinical use in humans. Thus, the validation of optimal isolating and culturing protocols is needed before the preparation of cell-based medicinal products for use in humans [16]; these products must further undergo in-depth functional characterization to confirm their safety and efficiency.

Similar effects to MSC-derived soluble factors may be also mediated by EVs carrying several bioactive molecules with a pro-regenerative capacity and regulating the biological functions of various target cells in injured tissues [17]. The results obtained during the TERCELOI clinical trial demonstrated that reiterative infusions of histocompatible BM-MSCs (derived from pediatric donors) can improve the bone parameters and quality of life of pediatric patients suffering from osteogenesis imperfecta (OI). Moreover, it has been indicated that MSC therapy elicited a pro-osteogenic paracrine response in patients enrolled in the clinical trial, confirming the efficiency of MSCs in the treatment of OI [18]. Importantly, because of the low immunogenicity of native, non-activated MSCs, they may be administrated into the injured tissue not only in an autologous but also in an allogeneic manner [19]. Due to the pleiotropic paracrine mechanism of action consisting of secretion of bioactive soluble factors and EVs as well as the direct differentiation potential, MSCs may play a pivotal role in regeneration of several injured tissue [20,21]. Thus, MSCs, which are widely used in multiple clinical trials worldwide, represent a promising tool for tissue regeneration.

Currently, there are 356 ongoing clinical trials for MSC-based therapies (retrieved from ClinicalTrial.gov on 18 June 2021; studies found for “mesenchymal stem cells”) for human diseases, including bone diseases [22], diabetes [23], multiple sclerosis [24], and cardiovascular disorders [25]. Some of these are in phase II and are aiming at the evaluation of the efficacy and safety of MSC-based therapy. However, the clinical use of MSCs is obstructed by several important limitations, including the naturally low number of MSCs in tissues, the invasiveness of tissue-harvesting procedures, and the low expansion potential observed by some investigators [26,27]. Moreover, the supplementation of medium for MSC culture with serum such as fetal bovine serum (FBS) or other animal-derived components has prompted safety concerns around the possible transmission of prions and viruses to the recipient of the cell therapy [28]. Besides the optimization of the manufacturing process, other logistic activities related to the cell-based product, including storage and transport conditions, should be also evaluated before the first in-human trials are carried out. All manufacturing actions must be validated and documented at the stage of early product development to ensure the control of product quality and the fulfillment of the standards detailed in The Tissue & Cell Directive (2004/23/EC) and/or Good Manufacturing Practice, Annex I. Despite the lack of legal regulations concerning the standardization of cell-based manufacturing processes, there are many recommendations and guidelines regarding quality assurance and the biological safety of such therapies, including the recommendations of ISCT for defining MSCs or the statement of the International Federation for Adipose Therapeutics and Science (IFATS) for the culture of AT-derived MSCs (AT-MSCs) [29]. Therefore, local regulatory authorities demand that the manufacturer determine the product’s critical quality attributes (CQA), in particular its lot-to-lot consistency, identify potentially unsafe impurities by performing the quality control of raw materials, perform sterility and endotoxin tests [30], etc. Thus, the optimization of the protocols used for the processing and ex vivo expansion of AT-MSCs, the adjustment of the dose of cells necessary for the intended use of a medicinal product, and the preparation of the final product formulation so that it does not impact on the cells’ biological activity and supports MSCs’ capacity for interaction/regeneration with the target tissue represent real challenges and influence the efficiency of cell-based therapies. Taken together, for a prospective manufacturer of MSC-based medicinal products, it is essential to: (1) standardize the manufacturing process and prepare standard operating procedures (SOPs), (2) conduct in-process quality control (QC) tests during the manufacturing of cell-based medicinal products, and (3) conduct the manufacturing process in a GMP facility.

In our study, we isolated MSCs from AT collected as lipoaspirate, which was determined as the most suitable source of MSCs because there are fewer ethical concerns around it and the collection procedure used may be relatively non-invasive for the patient (AT is often medical waste from various surgical procedures). Although BM-MSCs are considered to be the gold standard of MSCs in research and clinical applications, the invasive procedure used for BM harvesting and the lower yield of BM-MSC isolation (when compared to the MSCs isolated from AT), especially in older donors, makes this an important limitation for clinical applications [31,32]. Compared with embryonic stem cells (ESCs) or induced pluripotent stem cells (iPSCs), MSCs, despite their lower plasticity, do not cause the formation of teratomas and do not cause ethical concerns [33].

In the current study, using a process approach, we optimized the protocols for the processing and ex vivo expansion of AT-derived MSCs (AT-MSCs) for the development of Advanced Therapy Medicinal Products (ATMPs) for use in humans. We defined the QC parameters required for Hospital Exemption-ATMP (HE-ATMP) manufacturing and presented the initial specification of the final product containing QCA. Despite the lack of marketing authorization for HE-ATMP in the Member States of the European Union, these medicinal products are manufactured by industrial methods using good manufacturing practice (GMP) rules according to the individual order of a physician for their intended use.

## 2. Materials and Methods

### 2.1. Lipoaspirate Collection

Human adipose tissue (AT) was collected from healthy donors. AT from 11 human donors (age range: 26–54; nine females and two males; BMI range: 21.9–33.2) were used to perform all experimental stages in the process approach required in this study. Informed consent was obtained from all the subjects involved in the study. Approximately 50 mL of subcutaneous AT was obtained with a Body-Jet Liposuction device (Human Med AG, Schwerin, Germany). Then, the aspirated AT was filtered, washed, and collected using a FilterCollector (Corning, Tewskbury, MA, USA), from which it was retrieved with syringes (Becton Dickinson, BD; Franklin Lakes, NJ, USA). Lipoaspirate in syringes closed with Combi-Stoppers (B.Braun, Melsungen, Germany) were packed into a transport container equipped with a temperature recorder and subsequently transported at 2–8 °C to the laboratory. The isolation of stromal vascular fraction (SVF) cells was conducted on the same day as the tissue harvesting. The lipoaspirate collection procedure as well as research on isolated cells were conducted in accordance with the applicable regulations of human welfare and were approved by the local ethics committee (Bioethics Committee in the Regional Medical Chamber in Cracow, Poland; opinion no. 72/KBL/OIL/2017). According to the European Commission Directive 2006/17/WE of 8 February 2006, AT was harvested only from donors with negative results from lab screening for HIV, HBV, HCV, and syphilis. All raw materials and reagents were verified for their compliance to GMP rules for use in a manufacturing process and were delivered only by qualified producers/suppliers.

### 2.2. Isolation of SVF Cells

SVF cells were isolated from AT. Briefly, AT was washed with PBS solution w/o Ca^2+^, Mg^2+^ (HyClone, GE Healthcare Life Sciences, Malborough, MA, USA) and supplemented with 1% Antibiotic-Antimycotic Solution (ThermoFisher Scientific, Waltham, MA, USA). Subsequently, SVF cells were isolated by enzymatic digestion with collagenase NB6 GMP Grade (Serva Electrophoresis, Heidelberg, Germany) for 40 min (or 60 min) at 37 °C. During the optimization of the enzymatic digestion conditions, the following collagenase concentrations were tested: A1 (0.55 mg/mL), A2 (1.10 mg/mL), and A3 (1.65 mg/mL). The activity of the collagenase was stopped by the addition of PBS and the tubes with AT were cooled down during further centrifugation (370× *g*, 10 min, 16 °C). After centrifugation, the pellet containing SVF cells was resuspended in complete cell culture medium ((DMEM/F12 supplemented with 10% FBS (both from Sigma-Aldrich, Merck, Darmstadt, Germany) and 1% Penicillin-Streptomycin solution (Gibco, ThermoFisher Scientific)) during the selection of the optimal collagenase concentration or other tested media designed for the culture of MSCs under GMP conditions (described in Section 2.5 during the selection of the optimal culture medium), then passed through a 100-μm strainer (Corning) to remove the remaining veins or tissue debris, washed with PBS solution w/o Ca^2+^, Mg^2+^ (HyClone, GE Healthcare Life Sciences), collected in a new centrifuge tube, and centrifuged (350× *g*, 7 min, RT). Finally, the pellet containing SVF cells was resuspended in complete cell culture medium, seeded in culture flasks (Corning), at a density of 5000–11,000 cells/cm^2^, and further cultured under standard culture conditions (37 °C, 5% CO_2_, 95% humidity). Fresh culture medium was added after 2 days post-seeding. AT-MSCs were passaged with Tryple Select Enzyme (Gibco, ThermoFisher Scientific) when the confluence of cells reached close to 80–90% (at app. 4–5 day of culture). AT harvested from three individual human donors was used to replicate each step of the optimization of SVF isolation.

### 2.3. Modification of SVF Isolation Procedure by Adding the Step of Red Blood Cell Lysis

The standard optimized protocol for SVF isolation (described in Section 2.2) was compared to alternative protocols with the modification that it included a stage of red blood cell lysis using ZAPR™ Red Blood Cell Lysing Buffer (Incell, San Antonio, TX, USA). Briefly, after the first centrifugation (370× *g*, 10 min, 16 °C) and cell suspension filtration with a 100-μm strainer (Corning) to remove the remaining veins or tissue debris, 4 to 5 volumes of ZAPR™ Red Blood Cell Lysing Buffer were added to the cell suspension, mixed, and incubated for 5 min (Alternative Protocol no. 1, AP no. 1) or 10 min (Alternative Protocol no. 2, AP no. 2) at RT. Then, the cell suspension was diluted two times in complete cell culture medium and centrifuged (350× *g*, 7 min, 16 °C). The cell pellet was resuspended in complete culture medium and seeded in a culture flask (Corning) at a density of 0.1–0.15 × 10^4^ cells/cm^2^ and further cultivated under standard culture conditions (37 °C, 5% CO_2_, 95% humidity). The culture medium was changed every 2–3 days.

### 2.4. Cell Counting and Viability Assessment

#### 2.4.1. RD Phase

The number of SVF cells or AT-MSCs was counted using a Scepter^TM^ 2.0 Cell Counter (Millipore, Merck) equipped with Scepter Cell Counter Sensors 60 µm (Millipore, Merck). The viability of the cells was examined using a manual dye-exclusion method. The cell suspension was mixed (1:1) with 0.4% Trypan Blue solution (ThermoFisher Scientific) and further (after 2–4 min) counted in a Burker chamber using an Olympus IX81 microscope equipped with a MicroPublisher 3.3 RTV camera (Olympus, Tokyo, Japan).

#### 2.4.2. Implementation Phase

To evaluate the number and viability of SVF cells or AT-MSCs, the automated fluorescence cell counter ADAM-MC^TM^ (Nanoentek, Hwaseong-si, Gyeonggi-do, Korea), which utilizes precision disposable microchips (Nanoentek), was employed. Cells in suspension were stained with Accustain Solution T and Accustain Solution N (ADAM^TM^ AccuChip Kit, Nanoentek) and further applied to microchips according to the manufacturer’s protocol.

### 2.5. Culture of AT-MSCs

To select the optimal GMP-grade culture medium designated for MSC culture for clinical applications, directly after isolation, as described in Section 2.2, SVF cells were seeded in culture flasks and cultured in parallel in the following media: Medium A: αMEM supplemented with 10% human platelet lysate MultiPL’30 (both from Macopharma, Tourcoing, France), 2 IU/mL Heparinum WZF (Polfa S.A., Starogard Gdański, Poland), and 1% Penicillin-Streptomycin solution (ThermoFisher Scientific); Medium B: complete MSC NutriStem XF Medium (Biological Industries, Kibbutz Beit-Haemek, Israel) supplemented with 1% Penicillin-Streptomycin solution (ThermoFisher Scientific); Medium C: complete StemPro MSC SFM medium (ThermoFisher Scientific) supplemented with 1% Penicillin-Streptomycin solution (ThermoFisher Scientific). Cells were seeded at a density of 2 × 10^6^ per flask (175 cm^2^; Corning) and further cultured to assess their morphology, confluence, and yield in culture after passage nos. 1, 2, and 3. The medium was changed every 2–3 days.

To select the optimal culture flasks suitable for MSC culture for clinical applications, directly after the isolation described in Section 2.2, SVF cells were parallel seeded on the following Polystyrene Tissue Culture Flasks with Vented Caps manufactured by (1) Corning, (2) Eppendorf (Hamburg, Germany), or (3) TPP (Techno Plastic Products AG, Trasadingen, Switzerland) in the culture medium (selected based on the assessment described above): αMEM supplemented with 10% human platelet lysate MultiPL’30 (both from Macopharma), 2 IU/mL Heparinum WZF (Polfa S.A.), and 1% Penicillin-Streptomycin solution (ThermoFisher Scientific).

The morphology of AT-MSCs was visualized during cell culture using an Olympus IX81 microscope equipped with a MicroPublisher 3.3 RTV camera (Olympus). The yield of the culture (presented as the total number of cells detached from 1 cm^2^ of growth area) was evaluated using a Scepter^TM^ 2.0 Cell Counter (Millipore, Merck) according to the procedure described in Section 2.4. The viability of AT-MSCs was evaluated using the Trypan blue exclusion method, as described in Section 2.4. AT-MSCs isolated from AT harvested from three individual human donors were used to replicate the optimization of the cell culture.

### 2.6. Kinetics of AT-MSCs Growth

To assess the kinetics of the growth of AT-MSCs necessary for the estimation of the timeline of the manufacturing process used for cell-based medicinal products, AT-MSCs were seeded on cell culture flasks (Corning; selected during the evaluation described in Section 2.5) at concentrations of 3000, 4000, 5000, or 6000 cells per 1 cm^2^. The morphology and confluence of AT-MSCs were evaluated at day 2, 3, and 4 post-cell seeding using an Olympus IX81 microscope equipped with a MicroPublisher 3.3 RTV camera (Olympus).

### 2.7. Antigenic Phenotyping by Flow Cytometry 

To confirm the identity of the isolated AT-MSCs in accordance with the minimal criteria for defining multipotent MSCs published by the ISCT [3], the cells were re-suspended in standard staining medium (αMEM supplemented with 2% human platelet lysate MultiPL’30 (both from Macopharma) and 2 IU/mL Heparinum WZF (Polfa S.A.)) and further immunolabelled with the following monoclonal antibodies against the following human antigens: anti-CD45 (PE, clone: HI30, Biolegend, San Diego, CA, USA), anti-CD14 (FITC, clone: MφP9, BD Bioscences, Franklin Lakes, NJ, USA), anti-CD19 (FITC, clone: HIB19, BD Bioscences), anti-CD34 (PE, clone: 581, BD Bioscences), anti-CD31 (PE, clone: WM59), anti-CD44 (PE, clone: BJ18, Biolegend), anti-CD73 (PE, clone: AD2, Biolegend), anti-CD90 (PE, clone: 5E10, Biolegend), anti-CD105 (PE, clone: 43A3, Biolegend), and anti-HLA-DR (PE, clone: L243, Biolegend). For each analyzed antigen, appropriate isotype control was used as follows: mouse IgG1 (FITC, clone: MOPC-21, BD Bioscences), mouse IgG1 (PE, clone: MOPC-21, BD Bioscences), mouse IgG2a (PE, clone G155-178, BD Bioscences), and mouse IgG2b (FITC, clone: 27-35, BD Bioscences). Staining was performed according to the manufacturer’s protocols for 30 min at 4 °C. Cells were further washed with PBS w/o Ca^2+^, Mg^2+^ (HyClone, GE Healthcare Life Sciences) and analyzed using an BD LSR Fortessa flow cytometer and the FACS Diva software (Becton Dickinson, Franklin Lakes, NJ, USA).

### 2.8. Trilineage Differentiation of AT-MSCs

In the case of the osteogenic and adipogenic differentiation of AT-MSCs, 5.0 × 10^3^ cells or 1.0 × 10^4^ cells, respectively, were seeded per 1 cm^2^ in complete cell culture medium (αMEM supplemented with 10% human platelet lysate MultiPL’30 (both from Macopharma), 2 IU/mL Heparinum WZF (Polfa S.A.), and 1% Penicillin-Streptomycin solution (ThermoFisher Scientific)) and further cultured to obtain 60% of their confluence. Subsequently, the culture medium was replaced with a complete StemPro Osteogenesis Differentiation Kit or StemPro Adipogenesis Differentiation Kit (Gibco, ThermoFisher Scientific), stimulating osteogenic or adipogenic differentiation, respectively. The cultures were refed every 2–3 days.

To induce the chondrogenic differentiation of AT-MSCs, micromass cultures were generated by seeding 5-µL droplets of cell suspension (1.6 × 10^7^ viable cells/mL) and incubating them for 1 h under high-humidity conditions; further micromasses were flooded with complete culture medium αMEM supplemented with 10% human platelet lysate MultiPL’30 (both from Macopharma), 2 IU/mL Heparinum WZF (Polfa S.A.), and 1% Penicillin-Streptomycin solution (ThermoFisher Scientific). After 24 h, the medium was changed for StemPro Chondrogenesis Differentiation Medium (Gibco, ThermoFisher Scientific). The cultures were refed every 2–3 days.

Cells were examined for adipogenic differentiation at the 11th day of differentiation of the culture by direct microscope observation to identify oil droplets characteristic of adipogenesis. In the case of osteogenic and chondrogenic differentiation, cells were examined at 14 or 21 days of culture, respectively, following histochemical staining to identify the cell phenotype.

### 2.9. Chondrogenic Differentiation of AT-MSCs in Microenvironment Resembling Conditions in Human Joints

As described previously, chondrogenic differentiation was performed by seeding 5-µL droplets of cell suspension (containing 1.6 × 10^7^ viable cells/mL), which was further incubated under high humidity (under safety cabinet) for 1 h. Next, after 24 h of being cultured in αMEM medium supplemented with 10% human platelet lysate MultiPL’30 (both from Macopharma), 2 IU/mL Heparinum WZF (Polfa S.A.) and 1% Penicillin-Streptomycin solution (ThermoFisher Scientific) were exchanged for StemPro Chondrogenesis Differentiation Medium (Gibco, ThermoFisher Scientific) or fresh standard cell culture medium (αMEM supplemented with 10% human platelet lysate MultiPL’30 (both from Macopharma), 2 IU/mL Heparinum WZF (Polfa S.A.), and 1% Penicillin-Streptomycin solution (ThermoFisher Scientific)). Cells were cultured in the AVATAR System (Xcellbio, San Francisco, CA, USA), which allows one to mimic the microenvironment of the human joints by tuning the oxygen and pressure levels. Cells were cultured in the following conditions: (1) 1 PSI, 21% O_2_; (2) 1 PSI, 5% O_2_; (3) 2 PSI, 5% O_2_; or (4) 5 PSI, 5% O_2_. The cultures were refed every 2–3 days.

### 2.10. Histochemical Staining

At the 14th or 21st day of chondrogenic or osteogenic differentiation, respectively, the cells were washed with PBS (HyClone, GE Healthcare Life Sciences) and fixed with 4% paraformaldehyde (POCH, Avantor Performance Materials Poland) for 30 min at RT. To evaluate the calcium phosphate deposition, which appears around cells differentiated into osteoblasts, after fixation the cells were rinsed with distilled water, stained with 2% Alizarin Red Solution (Millipore, Merck) for 2–3 min, and subsequently washed with PBS (Hyclone, GE Healthcare Life Sciences). To visualize the sulfated proteoglycans, which appear during chondrogenic differentiation, fixed cells were rinsed with PBS (HyClone, GE Healthcare Life Sciences) and stained with Alcian-Blue Staining Solution (EMD Millipore, Merck) for 30 min. Subsequently, the cells were rinsed three times with 0.1 N HCl (POCH, Avantor Performance Materials Poland) and then distilled water was added to neutralize the acidity. After histochemical staining, the cells were visualized using an Olympus IX81 microscope equipped with a MicroPublisher 3.3 RTV camera (Olympus).

### 2.11. Validation of Manufacturing Process of HE-ATMP

According to the established in-process quality controls and acceptance criteria for manufacturing of HE-ATMP (Table 1), the validation of the manufacturing process of HE-ATMP (containing AT-MSCs as an active substance) as previously described in the internal standard operating procedures (SOPs), was conducted by properly trained lab technicians in the GMP facility (Cell & Tissue Culture Laboratory, Jagiellonian Center of Innovation in Krakow, Poland) to verify whether the final product (QCA are described in Table 2) met the proposed acceptance criteria. The manufacturing process was conducted in A and B air cleanliness classes (classified according to EN ISO 14644-1). During the whole aseptic process, the maximum permitted airborne particle concentration was monitored. Aseptic conditions during the validation process were monitored using methods such as surface sampling, settle plates, and volumetric air samples according to defined GMP SOPs. During the validation of the manufacturing process, the AT harvested from two individual human donors was used to manufacture three independent batches of the final product.

### 2.12. Stability Study of HE-ATMP

AT-MSCs were passaged as described in Section 2.2 and further washed with PBS w/o Ca^2+^, Mg^2+^ (ThermoFisher Scientific). Then, a single dose of the final product containing 10 × 10^6^ AT-MSCs resuspended in a carrier solution containing Ringer lactate (Fresenius Kabi, Bad Homburg, Germany) supplemented with 1.0% human albumin (CSL Behring, King of Prussia, PA, USA) and 2.5% glucose (Fresenius Kabi) were transferred to the selected container closure system (BD syringe with luer-lock tip; BD; closed with CombiStopper, B.Braun) and stored at 2–8 °C. The viability of the AT-MSCs was measured every 3 h, as described in Section 2.4 (implementation phase). 

### 2.13. Sterility Testing

During the validation of the manufacturing process of HE-ATMP for each manufactured batch, the sample of starting material and the sample of the final product underwent sterility testing using a direct inoculation method according to Ph. Eur. 2.6.1. The sterility tests were performed in the Polish Stem Cell Bank.

### 2.14. Endotoxins’ Testing

During the validation of the manufacturing process of HE-ATMP for each manufactured batch, the sample of the final product was tested using the LAL method to evaluate the level of bacterial endotoxins according to the Ph. Eur. 2.6.14. The endotoxins’ tests were performed in the Polish Stem Cell Bank.

### 2.15. Statistical Analysis

Data are represented as means ± standard deviations (SDs). Statistical analyses were performed using an ANOVA with Tukey’s post hoc test. *p* < 0.05 was considered statistically significant.

## 3. Results

### 3.1. SVF Cells Were Effectively Isolated from Adipose Tissue

**Figure 1:**
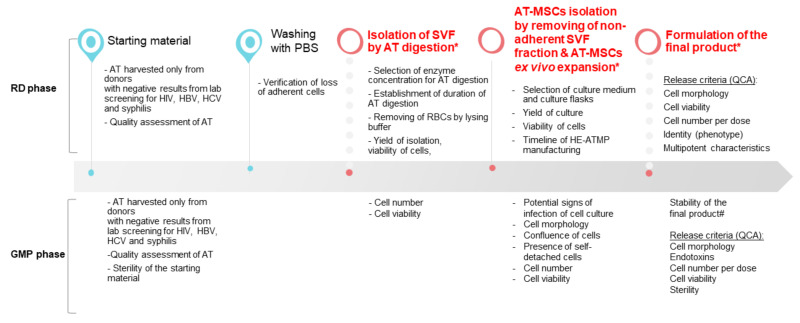
Overview of the HE-ATMP manufacturing process. In the diagram, we marked parameters that were assessed during the RD phase and the GMP phase (validation of the manufacturing process). (*) CPP: critical process parameters; (#) stability study was conducted during the validation of the manufacturing process; QCA: quality control attributes.

The designed experimental layout aimed at the optimization of the manufacturing process of HE-ATMP containing AT-MSCs as an active substance, which was divided into the following stages: (1) isolation of stromal vascular fraction (SVF) cells, (2) culture of AT-MSCs, and (3) termination of AT-MSC culture, and formulation of the final cell-based product followed by quality control (QC) tests of the released final product (Figure 1). The critical process parameters (CPP) were identified (Figure 1) and a number of experiments were conducted to select the optimal experimental conditions.

**Figure 2:**
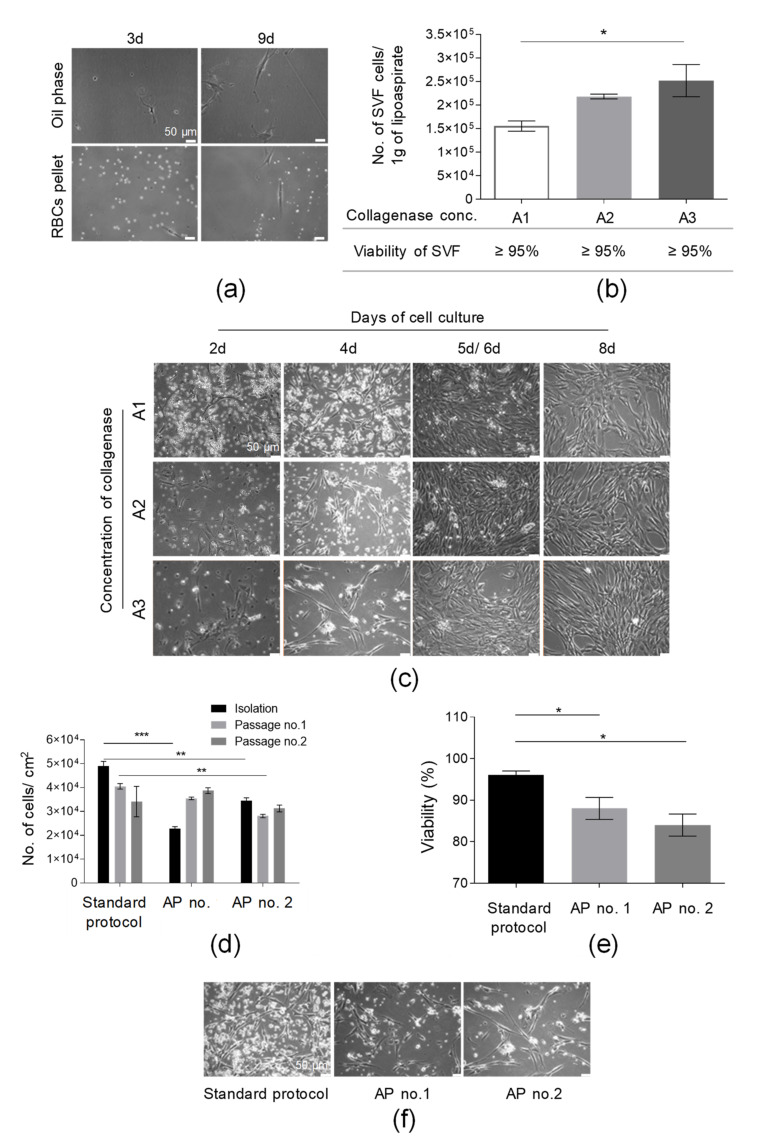
Optimization of the procedure of isolation of SVF cells from adipose tissue (AT). (**a**) Washing of AT with PBS did not cause the loss of adherent cells of SFV. Representative images of a few cells isolated from PBS obtained after the washing of AT 3 and 9 days post-seeding. PBS were centrifuged (300× *g*, 5 min, RT); the oil phase and, subsequently, pellet containing RBCs and tissue sections were resuspended in complete culture medium (DMEM/F12 supplemented with 10% FBS and 1% Penicillin-Streptomycin solution) and cultured for 9 days. (**b**) Examination of the suitability of selected collagenase concentrations for AT digestion. Yield of isolation is presented as the total number of SVF cells obtained per 1 g of lipoaspirate. Viability of SVF cell is presented in the table. AT was incubated with A1, A2, or A3 concentrations of collagenase for 40 min at 37 °C and, after a washing step, the number of cells isolated was counted. (**c**) Establishment of primary culture for analyzed collagenase concentrations. SVF cells isolated from AT following digestion with A1, A2, or A3 collagenase concentrations were seeded on culture flasks in complete culture medium (DMEM/F12 supplemented with 10% FBS and 1% Penicillin-Streptomycin solution) and assessed by light microscopy at days 2, 4, and 8 (for A1, A2, A3 collagenase concentrations); day 5 (for A1 and A2 collagenase concentrations); or day 6 (for A3 collagenase concentration). (**d**) Examination of the influence of an additional step of RBC lysis on the yield of the cell isolation and culture (presented as the number of isolated cells or cells detached from 1 cm^2^ of cell growth area) directly after SVF isolation and two consecutive passages. (**e**) Effect of RBCs’ lysis on SVF viability. Lysis of RBCs was conducted according to AP no. 1 or 2. (**f**) Morphology of adherent cells isolated using standard SVF isolation protocol and two alternative protocols (AP no. 1 or 2). Representative pictures were taken prior to the first passage. Scale bars: 50 µm. Results (**b**,**d**,**e**) are presented as means ± SDs. *n* = 3 (biological replicates, corresponding to three individual human donors) and they were assessed by an ANOVA model with Tukey’s post hoc test. The *p* values less than 0.05 (*p* < 0.05) were considered to be significant and labeled by an asterisk *p* < 0.05 (*), *p* < 0.01 (**), *p* < 0.001 (***).

In the first step of the isolation of SVF cells, lipoaspirate was washed with PBS solution supplemented with antibiotics and antimycotic solution to remove red blood cells (RBCs). It was shown that the washing of AT with PBS solution did not cause the loss of a significant amount of adherent cells in both fractions (oil phase and PBS with RBCs) removed from a tube containing AT following the washing stage (Figure 2a). At 9 d post-seeding in the oil phase and RBC pellet on culture flasks, we observed only a few adherent cells per microscopic field (Figure 2a). This step may also allow us to remove accidental contamination (e.g., with bacteria or fungi) to prevent the contamination of the manufacturing environment.

To select the optimal collagenase concentration suitable for effective AT digestion, the yield of isolation of SVF cells was calculated as the number of SVF cells isolated from 1 g of used lipoaspirate. The obtained results indicate that the greatest number of isolated SVF cells (2.52 × 10^5^ ± 0.34 × 10^5^/1 g of lipoaspirate) were found after digestion with an A3 collagenase concentration; subsequently, a slightly lower yield of SVF isolation was observed for an A2 collagenase concentration (2.18 × 10^5^ ± 0.05 × 10^5^/1 g of lipoaspirate), as presented in Figure 2b. We observed a statistically significant higher yield of SVF isolation following AT digestion with an A3 collagenase concentration when compared to digestion with an A1 collagenase concentration. Although no impact of the used collagenase concentration on the viability of isolated SVF cells was detected (Figure 2b), we observed the lowest cell growth rate of isolated adherent cells following digestion with an A3 collagenase concentration, especially up to 5 d post SVF seeding. Interestingly, extending the time of the enzymatic digestion from 40 to 60 min for three tested collagenase concentrations did not result in increasing the yield of SVF isolation (Figure S1a), confirming the total digestion of AT within 40 min. Importantly, the A2 collagenase concentration was selected for further experiments.

Subsequently, the yields of the SVF isolation and AT-MSC culture for a standard SVF isolation protocol (described in Section 2.2) were compared to the yield of the SVF isolation and AT-MSC culture obtained following SVF isolation with alternative protocols of SVF isolation (AP nos. 1 and 2, containing the step of red blood cells lysis; see Section 2.3). The greatest number of isolated SVF cells which also exhibited the highest viability was obtained for the standard SVF isolation protocol when compared to alternative protocols AP nos. 1 and 2 (Figure 2d,e). AP no. 1 allowed us to isolate adherent cells that exhibited an exponential increase in yield of culture (presented as the total number of cells detached from 1 cm^2^ of cell growth area) over the course of time (Figure 2d) when compared to cells obtained according to the AP no. 2 protocol.

Moreover, it was also shown that the cells isolated with the standard protocol exhibited the greatest adherence capacity when compared with cells isolated with alternative protocols AP nos. 1 and 2 (Figure 2f). Thus, the washing of AT with PBS solution represents an efficient and safe step of RBC removal and the additional step of RBC lysis was not included in the protocol of SVF isolation. To summarize, the stage of isolation of SVF cells was successfully optimized.

### 3.2. AT-MSCs Were Efficiently Cultured in In Vitro Conditions in Accordance with GMP Requirements

**Figure 3:**
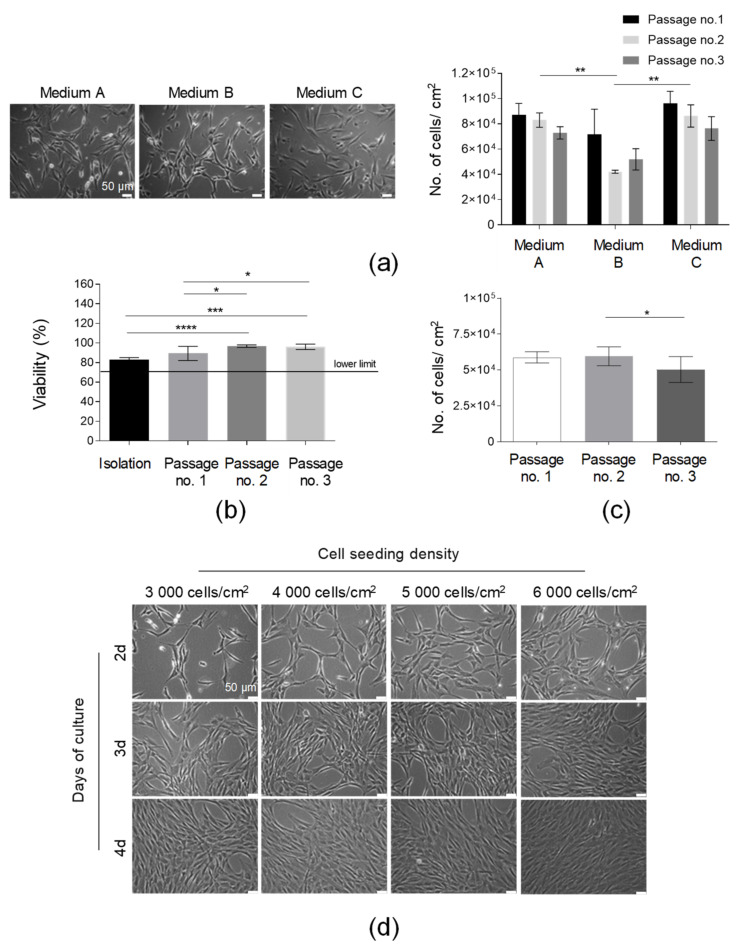
Optimization of the culture conditions of AT-MSCs. (**a**) Selection of the optimal medium for AT-MSC culture. Morphology of the AT-MSCs cultured in the following culture media: Medium A, Medium B, and Medium C. Efficiency of the AT-MSC culture in tested media presented as the number of cells detached from 1 cm^2^ of the growth area at passages nos. 1, 2, and 3. (**b**) Analysis of cell viability from the isolation to the third passage. (**c**) Analysis of the number of AT-MSCs obtained in three consecutive passages. (**d**) Kinetics of the growth of AT-MSCs seeded on cell culture flasks at concentrations equal to 3000, 4000, 5000, or 6000 cells per 1 cm^2^ at days 2, 3, and 4 post-cell seeding by light microscopy. Scale bars: 50 µm. Results (**a**–**c**) are presented as means ± SD. N = 3 (biological replicates, corresponding to three individual human donors), and they were assessed by an ANOVA model with Tukey’s post hoc test. The *p* values less than 0.05 (*p* < 0.05) were considered to be significant; *p* < 0.05 (*), *p* < 0.01 (**), (*), *p* < 0.001 (***), *p* < 0.0001 (****).

After the standardization of the SVF isolation protocol, AT-MSCs’ culture optimization was subsequently conducted. Firstly, the effect of the culture media on the AT-MSC morphology and growth kinetics was examined. All tested media (Medium A, Medium B, and Medium C) were animal component-free and designated for MSC culture for clinical applications. Our results indicated that the isolated adherent fraction of SVF cells represented spindle-shaped elongated cells possessing a fibroblast-like morphology in all the tested media (Figure 3a). The AT-MSCs cultured in Medium A and Medium C demonstrated a comparably high yield of cell growth (presented as the number of cells detached from 1 cm^2^ of cell growth area) over the consecutive three passages, whereas the yield of the cell culture in Medium B was the lowest, especially after the second and third passages. Thus, Medium A was selected for further AT-MSC culturing. Finally, we compared the confluence and number of AT-MSCs obtained in culture flasks (75 cm^2^) produced by three different manufacturers. The number of cells obtained from the three compared flasks delivered by the different vendors was comparable over the consecutive three passages (Figure S1b). Thus, all the tested culture flasks may be used for the culture of MSCs. However, during the selection of the most prominent flasks, it is also necessary to compare the certificates of analysis (CoA) delivered by manufacturers.

To determine the timeline of the HE-ATMP manufacture and confirm the in-process QC limits, we analyzed (1) the AT-MSC viability, (2) the yield of the AT-MSC culture, and (3) the confluence of the AT-MSCs depending on the cell seeding density at consecutive days of culture. The viability results confirmed that the AT-MSCs cultured in Medium A on passages 1–3 exhibited a viability of over 85% (Figure 3b). This result was above our accepted lower QC limit, which was ≥70% (Table 1). The number of AT-MSCs detached from 1 cm^2^ of cell growth area was comparable between three consecutive passages (Figure 3c). The analysis of the cell seeding density showed that AT-MSCs reached a 90% confluence at day 3 when seeded at a density of 6 × 10^3^/1 cm^2^ or at day 4 when seeded at a lower density equal to 4 × 10^3^/1 cm^2^ (Figure 3d). Such analysis allowed lab technicians working in the GMP facility to design a timeline of the manufacturing process to release the final product at the established day of the product application. Taken together, Medium A was selected for further AT-MSC culture. Importantly, AT-MSCs growing in such medium exhibited a proper morphology and proliferation rate and it was possible to manufacture a few doses of the final product from one starting material (AT).

### 3.3. The Identity of Isolated AT-MSCs Was Confirmed According to ISCT Recommendations

**Figure 4:**
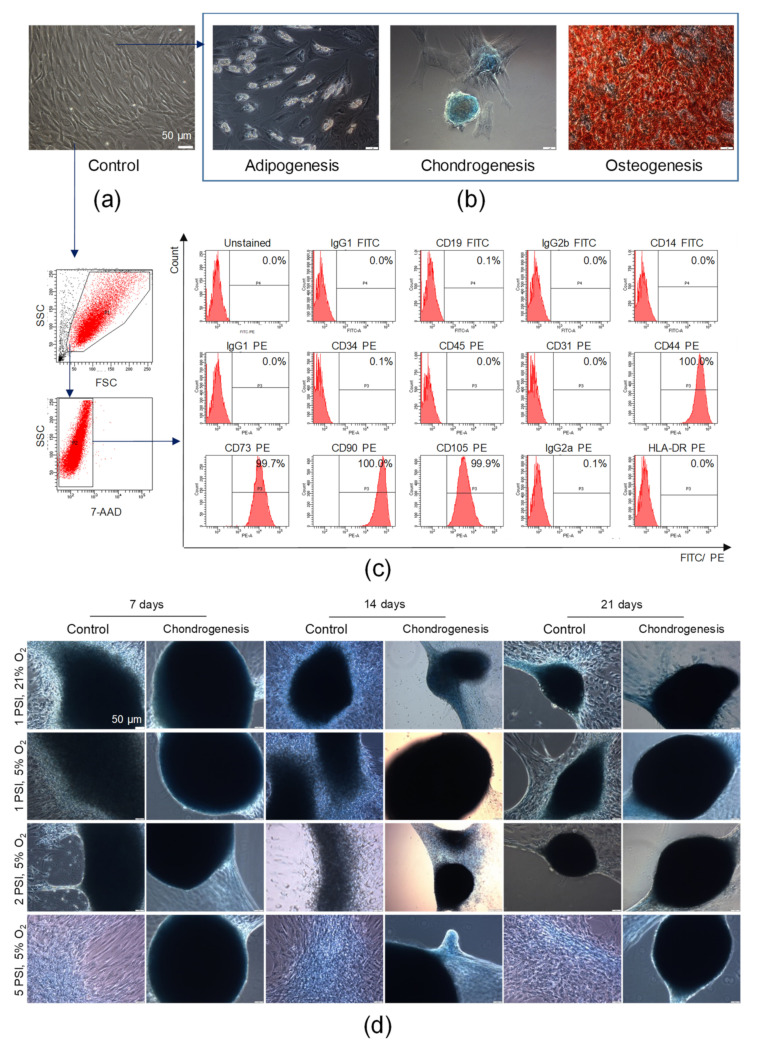
Characterization of AT-MSCs according to the criteria defining multipotent MSCs proposed by the International Society for Cellular Therapy and their chondrogenic potential. (**a**) Representative images of the morphology of AT-MSCs at passage 4 by light microscopy. (**b**) Trilineage differentiation potential of AT-MSCs. Representative images of AT-MSCs differentiated into osteoblasts, chondroblasts, and adipocytes. AT-MSCs were cultured using a StemPro Osteogenesis Differentiation Kit (for 21 days), StemPro Chondrogenesis Differentiation Kit (for 14 days), or StemPro Adipogenesis Differentiation Kit (for 11 days). Post differentiation, AT-MSCs were fixed with paraformaldehyde and stained with Alizarin Red S (red staining of deposits of calcium phosphate characteristic for osteogenic differentiation) or Alcian Blue (blue staining of sulfated proteoglycans characteristic for chondrogenic differentiation), whereas oil droplets characteristic for adipogenic differentiation were shown in unfixed and unstained AT-MSCs. Scale bars: 50 µm. (**c**) Antigenic profile of AT-MSCs by flow cytometry. Representative histograms of the expression of MSC-negative markers (CD19, CD14, D45, CD34, CD31), MSC-positive markers (CD44, CD73, CD90, CD105), and HLA-DR antigens on viable (7-AAD^−^) AT-MSCs. (**d**) Chondrogenic differentiation potential of AT-MSCs in a microenvironment resembling the conditions in human joints. Representative images of AT-MSCs cultured using the StemPro Chondrogenesis Differentiation Kit or standard cell culture medium (Control) in a hypoxic (5% O_2_) or normoxic (21% O_2_) environment parallel exposed to normal (1 PSI) or high (2 or 5 PSI) pressure in the Avantar System. At 7, 14, and 21 days, AT-MSCs were fixed with paraformaldehyde and stained with Alcian Blue. Scale bars: 50 µm.

We found that AT-MSCs exhibited adhesion to the polystyrene surfaces of cell culture flasks when maintained in standard culture conditions in vitro. AT-MSCs are spindled-shaped cells that are fibroblast-like in morphology (Figure 4a). To confirm the mesodermal origin of AT-MSCs, an analysis of their trilineage differentiation potential was conducted. For such purpose, AT-MSCs were differentiated into chondroblasts or osteoblasts for 14 or 21 days, respectively, whereas AT-MSCs were differentiated into adipocytes for 11 days in tissue-specific differentiation media. We found that AT-MSCs exhibited trilineage differentiation potential (as shown in Figure 4b), which also confirmed their MSC phenotype, as defined by the minimal criteria recommended by the ISCT [3]. By using a flow cytometry platform, we subsequently demonstrated that our population of isolated adipose tissue-derived cells exhibited a high expression of MSC-specific markers such as CD44, CD73, CD90, and CD105 and did not express markers of hematopoietic cells such as CD45, CD14, CD19, and CD34; endothelial cell-specific marker CD31; or HLA-DR antigen (Figure 4c). Thus, based on the observed ability to adhere to plastic surfaces, the trilineage differentiation potential, and the characteristic antigenic profile, we confirmed the identity of the isolated adipose tissue-derived cells previously described as AT-MSCs, representing a subpopulation of MSCs.

Subsequently, to evaluate whether the microenvironment mimicking the conditions in the human joints impacted on the chondrogenic potential of AT-MSCs, AT-MSCs were cultured in the AVATAR system. The obtained results indicated that the AT-MSC culture conditions, particularly under a high pressure of 2 PSI and 5 PSI in hypoxia in culture medium dedicated to chondrogenic differentiation (StemPro Chondrocyte Differentiation Kit), affected changes in cellular morphology. High pressure (2 PSI and 5 PSI) and hypoxia impact on the aggregation of cells, which start to form micromasses characteristic of chondrogenic differentiation in vitro. Furthermore, the AT-MSCs cultured in chondrogenic differentiation medium under a high pressure formed the largest and the most solid micromasses compared to the control conditions (cells cultured in standard culture medium aMEM supplemented with 2% human platelet lysate MultiPL’30). Representative images after 7, 14, and 21 days of AT-MSC culture are presented in Figure 4d.

### 3.4. Validation of Manufacturing Process of HE-ATMP

Based on the results obtained, firstly, we defined in-process QC tests including acceptance criteria and subsequently prepared the standard operating procedures (SOPs) for the manufacturing of HE-ATMP containing AT-MSCs as an active substance. The defined QC parameters are presented in Table 1. The manufacturing process was divided into the following stages: (1) isolation of SVF cells; (2) culture of AT-MSCs; and (3) termination of AT-MSCs culture and formulation of the final product. The parameters examined during the stage of SVF cell isolation (stage I) include the cell number (number of isolated SVF cells), their viability, and the sterility of the starting material. During the stage of culture of AT-MSCs as the adherent fraction of SVF (stage II) as well as the termination of the AT-MSC culture (stage III), we identified the following parameters of QC: the presence of signs of potential infection of cell culture, cell morphology and confluence, the presence of self-detached cells, cell number, and cell viability. Moreover, we also defined the specification of the final product (containing QCA), which is presented in Table 2.

Based on the defined QC tests, the designed specification of the final product, and the prepared SOPs containing protocols for the isolation and culture of AT-MSCs, the validation of the manufacturing process of HE-ATMP was conducted in the GMP facility. The obtained batch analysis data for validation series are presented in Table 3. The obtained results confirmed that the manufactured cell-based products met all the criteria listed in the specification (Table 2).

**Figure 5:**
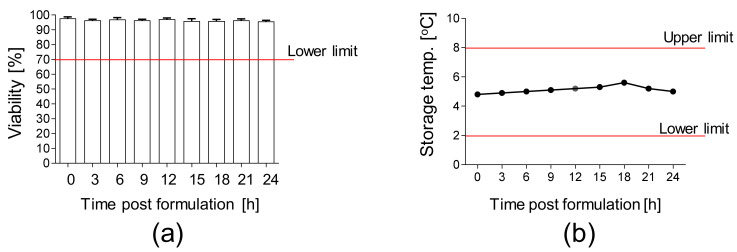
Stability of AT-MSCs solution. (**a**) Viability of AT-MSCs post-product formulation. A total of 10 × 10^6^ AT-MSCs were resuspended in carrier solution, transferred into container closure system, and stored at 2–8 °C. Viability of AT-MSCs was measured every 3 h up to 24 h post-product formulation by an automated cell counter ADAM. (**b**) Storage temperature of AT-MSC solution during the stability test. Results are presented as means ± SDs, *n* = 3 (independent validation batches).

Moreover, the stability of the final product packed in the container closure system and subsequently stored at 2–8 °C was analyzed. We confirmed a high viability (>90%) of AT-MSCs up to 24 h after the final product formulation (Figure 5a) at the temperature of 2–8 °C (Figure 5b).

## 4. Discussion

According to the European Medicines Agency (EMA) regulatory framework, “advanced therapy medicinal products (ATMPs) are medicines for human use that are based on genes, tissues or cells” providing “groundbreaking new opportunities for the treatment of disease and injury”. Within types of ATMPs, we can distinguish, among others, tissue-engineered medicines, which “contain cells or tissues that have been modified so they can be used to repair, regenerate or replace human tissue” (https://www.ema.europa.eu). Due to the excellent biological properties of MSCs, including their wide differentiation potential, paracrine activity, and immunomodulatory potential, MSCs represent an ideal candidate for medicinal products for the treatment of tissue injury [8]. Thus, the major goal of the current study was to optimize the protocols for the processing and ex vivo expansion of AT-MSCs for the development of ATMPs for use in humans.

By employing a process approach, we designed protocols for the isolation and ex vivo expansion of AT-MSCs according to GMP requirements and applicable legal regulations. Subsequently, we conducted the optimization of the isolation and culture of AT-MSCs and transferred the developed methods from the RD laboratory to the manufacturing site (GMP facility). The whole process of isolation and ex vivo expansion of AT-MSCs was divided into the following stages: release of the starting material (AT) to the manufacturing process, processing of AT to isolate of SVF cells, expansion of AT-MSCs, and the termination of the culture and formulation of the final product (HE-ATMP). 

First, in collaboration with a surgeon who performs liposuction procedures, we established a method for the collection of AT that will ensure the sterility of the harvested biological material and will not reduce the viability of cells by applying a high negative pressure during the tissue collection. Next, we prepared sets dedicated for the collection and transport of AT. ATs were transported at the temperature of 2–8 °C, which allowed us to maintain the optimal tissue quality for the isolation of SVF cells and the expansion of AT-MSCs [34].

At the beginning of the AT processing, lipoaspirate was washed with PBS supplemented with a mixture of antibiotics and antimitotic solution to remove RBCs and any accidental contamination (to protect the manufacturing environment from bacterial/fungal contamination). In our study, RBCs were, in large part, removed following AT washing with PBS. The additional step of the removal of RBCs with lysing buffer (ZAPR™ Red Blood Cell Lysing Buffer) allowed us to remove the remaining RBCs but also decreased the viability of the SVF cells and their adherence to plastic culture surfaces, as well as the yield of the isolation of SVF and further AT-MSCs. It has been shown by other investigators that the use of lysing buffer may decrease the rate of proliferation of AT-MSCs [35]. To ensure the high biological potential of AT-MSCs, we resigned from the additional step of RBCs’ lysis. Subsequently, we confirmed that the A2 collagenase concentration (during a 40-min digestion) enabled the effective isolation of SVF cells to maintain their high viability. Collagenase-based AT digestion was shown to be the most effective in terms of cell recovery when compared to the mechanical isolation of SVF cells [36]. AT-derived SCs, including MSCs, tend to be localized in these perivascular niches [37]. In the case of mechanical methods of isolation, the disruption of the extracellular matrix was significantly reduced compared to enzymatic methods, resulting in many of the desired cells being left in larger tissue fragments, which were subsequently discarded [38].

During the next stage, we selected the following optimal cell culture medium: basal medium αMEM supplemented with human platelet lysate MultiPL’30 (PL) dedicated to the culture of MSCs for clinical application. It was shown that PL increases the doubling of the human BM-MSC population (from passages 1 to 3) and that the cells are more spindle shaped, are more elongated, and have denser cell bodies than MSCs from FBS-supplemented cultures. Moreover, PL does not affect the BM-MSC immunophenotype, trilineage differentiation potential, immunomodulatory properties, relate telomere length, or chromosomal stability when compared to FBS-supplemented cultures [39,40]. During the selection of manufacturer of PL, it is necessary to verify the quality specification of the PL according to the aspects presented by Oeller et al. [41]. Although on the market there are a few chemically defined media [42] and some of them were tested during our optimization research, we did not select one of them for use in our manufacturing process to simplify work (minimize the amount of manipulation it was necessary to conduct) in the cleanroom and to slightly reduce the manufacturing costs. To ensure the high quality of AT-MSCs, as the active substance of HE-ATMP, we conducted numerous cell seeding density assays to design a timeline of the manufacturing process. This approach will allow us to release the final product at an established day of the medicinal product application.

The identity of the AT-derived cells isolated as MSCs was confirmed according to the ISCT recommendations [3]. Moreover, we also confirmed the capacity of the AT-MSCs to differentiate into chondroblasts/chondrocytes in the microenvironment mimicking the conditions in human joints, which confirmed the intended use of the designed HE-ATMP product. Importantly, this particular product optimized in this study was designed for applications in cartilage and bone injuries. However, it should always be considered that, depending on the desired use of MSCs in distinct tissue injury treatments, such protocols would need to be set up and specifically optimized to obtain MSCs with the most optimal biological properties and therapeutic potential. This should be considered especially in the context of the immunomodulatory properties of MSCs cultured in different microenvironments. Although native MSCs predominantly exhibit anti-inflammatory properties [8,9,10], it has been shown that, in the presence of pro-inflammatory cytokines, they may accommodate pro-inflammatory features [43]. This phenomenon may depend on their microenvironment. For example, MSCs induced with low IFN-γ concentrations adopt a pro-inflammatory phenotype and lead to the activation of naive CD4^+^ T cells and the induction of CD8^+^ T cells in vitro and in vivo, while high IFN-γ levels downregulate HLA-DR expression in MSCs [43]. An increased expression of HLA-I antigens in IFN-γ-treated MSCs has also been shown; however, their surface expression was further downregulated via endocytosis. This phenomenon, called the “plasticity of MSCs”, relies on regulating the surface expression of HLA-I antigens to allow them to elicit a weaker immune response [15]. Moreover, Waterman et al. demonstrated that MSCs primed with the TLR4 agonist (e.g., lipopolysaccharide) adopted a pro-inflammatory phenotype, and they produced mediators able to induce T lymphocyte activation [44]. In contrast, MSCs primed with the TLR3 agonist (e.g., poly(I:C)) adopted an immunosuppressive phenotype expressing factors known to play a key role in the T cell-inhibiting effects of MSCs [44]. Interestingly, Lombardo et al. demonstrated that the activation of TLRs 2, 3, 4, and 9 on human AT-MSCs induced molecules in the nuclear factor κB (NF-κβ) pathway, resulting in the better engraftment and survival of these cells in inflammatory conditions or injured tissues [45]. In such circumstances, MSCs may accommodate pro-inflammatory or anti-inflammatory phenotypes, which needs to be carefully considered when these cells are propagated for clinical applications. Thus, the process of the optimal isolation and culturing is required prior to the preparation of MSC-based medicinal product for specific use in humans [16], depending on the tissue and the type of injury.

Based on the results obtained during RD studies, in-process QCs and acceptance criteria for the manufacturing of HE-ATMP were established. During the first stage of manufacturing, the following parameters were verified: sterility of starting material (AT) and number and viability of isolated SVF cells. During the second and third stages, we verified the presence of any signs of potential infection of cell culture, cell morphology and confluence, the presence of self-detached cells, the cell number, and the cell viability. Importantly, in the specification of the final product, HE-ATMP, we included the following QCA: cell morphology, endotoxins, cell number per dose, cell viability, and the sterility of the final product. In our study, HE-ATMP was designed and the validation of the manufacturing process was conducted to prepare the manufacturing site and manufacture the prototype of the further AMTP product to be tested during the clinical trials planned in the project. However, the number of tests necessary to conduct for a product for clinical trial (Advanced Therapy Investigational Medicinal Product, ATIMP) is much greater than that for HE-ATMP, as described by Le Chanteur et al. [46].

Besides the optimization of the processing and ex vivo expansion of AT-MSCs, we also prepared the protocols for the formulation of the final product and selected the carrier solution for these cells. Subsequently, during a stability study we confirmed the high viability of the AT-MSC solution packed in the container closure system up to 24 h post-product formulation. This assay allowed us to estimate the expiration date of the product (24 h). In the case of a cell-based medicinal product, the viability of cells represents a major parameter that influences the outcomes of patients after the administration of the medicinal product. During the formulation of the final product, AT-MSCs were resuspended in a carrier solution containing, among other things, human serum albumin (HSA) acting as a rich nutrient source ensuring the high viability of AT-MSCs. It has been shown that MSCs resuspended in Ringer solution supplemented with 1% HSA exhibit a high viability up 24 h post-product formulation. Similar results were observed for AT-MSCs resuspended in Ringer lactate or Hypo Thermosol when compared to AT-MSCs resuspended in only 0.9% NaCl. Thus, the type of carrier solution used represents an important factor that influences the maintenance of stability of MSC-based medicinal products [47]. In our approach, AT-MSCs do not undergo a cryopreservation procedure (except for the preparation of reference samples of the final product) in relation to other investigators, who added a cryopreservation step to the manufacturing procedure, as summarized by Mazini et al. [48].

In the manufacturing site, the validation of the manufacturing process of HE-ATMP containing AT-MSCs as an active substance was successfully conducted. The three manufactured validation batches fulfilled the release criteria included in the specification of the final product (HE-ATMP), confirming the good preparation of the SOPs throughout the whole manufacturing process. The exemplary SOPs for the isolation and culture of AT-MSCs from fat pads in the GMP facility were presented by Aghayan et al. [49]. To ensure the standardization of the manufacturing process of ATMP, some investigators have introduced automatic closed systems, such as the CliniMACS Prodigy Adherent Cell Culture System [50]. This solution may be effectively used for the large-scale manufacturing of MSCs, e.g., in the case of allogenic applications. In the case of autologous application, the manufacture of HE-ATMPs in open systems represents a less cost-effective approach.

To summarize, in the current study we presented a process approach leading to the optimization of the processing and ex vivo expansion of AT-MSCs. We identified CPPs and conducted multiple experiments to propose the optimal mode of action during the manufacturing process of cell-based medicinal products. Moreover, we defined in-process QCs and their acceptance criteria. To ensure the high quality and safety standards of cell-based medicinal products for clinical use, the manufacturing process must be accomplished in certified facilities following SOPs. The designed manufacturing process of HE-ATMP, which served as a prototype of an ATMP product in the clinical trial, was successfully validated in the manufacturing site.

## Figures and Tables

**Table 1:** In-process quality controls and acceptance criteria for the manufacturing of HE-ATMP. During the manufacturing process, we distinguished the following CPPs: the isolation of SVF cells, the culture of AT-MSCs, and the formulation of the final product.

**Stage I: Isolation of SVF Cells**			
**Parameter**	**Correct Result**	**Incorrect Result**	**Method**
(1) Cell number	Presence of SVF cells (successful isolation)	Lack of SVF cells (unsuccessful isolation)	Propidium iodide staining method combined with advanced image analysis (ADAM-MC^TM^ automated cell counter)
(2) Cell viability	≥60% → seeding SVF cells at a density 5000–11,000 cells/cm^2^	<60% → decrease in seeding growth area	Propidium iodide staining method combined with advanced image analysis (ADAM-MC^TM^ automated cell counter)
(3) Sterility of the starting material	Sterile → culture of cells	Non-sterile → utilization of cell culture	Direct inoculation (BD Bactec FX400 system)
**Stage II: Culture of AT-MSCs**			
**Parameter**	**Correct Result**	**Incorrect Result**	**Method**
(1) Potential signs of infection of cell culture	Orange or red-raspberry clear culture medium → culture of cells	Yellow and/or turbid culture medium → evaluation of cell morphology	Macroscopic observation, light microscopy
(2) Cell morphology	Characteristic for MSCs (spindle-shaped, elongated cells possessing fibroblast-like morphology)	Morphology different than characteristic for MSCs → utilization of cell culture	Light microscopy
(3) Confluence of cells	≥60% → passage of AT-MSCs <60% → further cell culture	<10% → evaluation of presence of self-detached cells	Light microscopy
(4) Presence of self-detached cells (qualitative assessment)	Lack of self-detached cells	Presence of a high amount of self-detached cells → utilization of cell culture	Light microscopy
(5) Cell number	At least 2× higher than number of seeding cells → continuation of manufacturing process	Less than 2× higher than number of seeding cells → utilization of cells	Propidium iodide staining method combined with advanced image analysis (ADAM-MC^TM^ automated cell counter)
(6) Cell viability	≥70% → continuation of the manufacturing process	<70% → utilization of cell culture	Propidium iodide staining method combined with advanced image analysis (ADAM-MC^TM^ automated cell counter)
**Stage III: Termination of AT-MSCs Culture and Formulation of the Final Product**			
**Parameter**	**Correct Result**	**Incorrect Result**	**Method**
Parameters (1)–(4) from Stage II: Culture of AT-MSCs			
Cell number	12 × 10^6^ viable cells	>12 × 10^6^ viable cells → the final product is not released	Propidium iodide staining method combined with advanced image analysis (ADAM-MC^TM^ automated cell counter)
Cell viability	≥70% → preparation of the final product	<70% → the final product is not released	Propidium iodide staining method combined with advanced image analysis (ADAM-MC^TM^ automated cell counter)

Conduction of the tests listed in the specification of HE-ATMP.

**Table 2:** Quality specification of the HE-ATMP. The parameters included in the table are the QCA of the final cell-based product.

Specification of the HE-ATMP			
Parameter	Limit or Range	Method	Additional Information
(1) Cell morphology	Characteristic for MSCs (spindle-shaped, elongated cells possessing fibroblast-like morphology)	Light microscopy	Parameter tested before the last passage
(2) Endotoxins	<2.5 UI/mL	LAL method (Endosafe^®^-PTS™ system)	Parameter tested before the last passage; the product is released before completion of the test
(3) Sterility of the final product	Sterile	Direct inoculation (BD Bactec FX400 system)	Sample is collected at the last passage and after the final product formulation; because of the short life-time of the product, the product is released before completion of the test
(4) Cell number per dose	10 × 10^6^ viable cells	Propidium iodide staining method combined with advanced image analysis (ADAM-MC^TM^ automated cell counter)	Parameter tested after the last passage
(5) Cell viability	≥70%	Propidium iodide staining method combined with advanced image analysis (ADAM-MC^TM^ automated cell counter)	Parameter tested after the last passage

**Table 3:** Batch analysis for three validation series of HE-ATMP.

Validation of Manufacturing Process of HE-ATMP		
Parameter	Limit or Range	Results
(1) Cell morphology	Characteristic for MSCs (spindle-shaped, elongated cells possessing fibroblast-like morphology)	Batch no. 1: Correct
		Batch no. 2: Correct
		Batch no. 3: Correct
(2) Endotoxins	<2.5 UI/mL	Batch no. 1: Correct
		Batch no. 2: Correct
		Batch no. 3: Correct
(3) Sterility of the final product	Sterile	Batch no. 1: Sterile
		Batch no. 2: Sterile
		Batch no. 3: Sterile
(4) Cell number per dose	10 × 10^6^ viable cells	Batch no. 1: 10 × 10^6^ viable cells
		Batch no. 2: 10 × 10^6^ viable cells
		Batch no. 3: 10 × 10^6^ viable cells
(5) Cell viability	≥70%	Batch no. 1: 98%
		Batch no. 2: 96%
		Batch no. 3: 98%

## Data Availability

The data supporting reported results can be found in repository at the Jagiellonian University in Krakow (Poland). The data are not publicly archived.

## Supplementary Materials

The following are available online at https://www.mdpi.com/article/10.3390/cells10081908/s1: Figure S1: Optimization of isolation and culture of AT-MSCs.
