# Supplementary material for: Processing and Ex Vivo Expansion of Adipose Tissue-Derived Mesenchymal Stem/Stromal Cells for the Development of an Advanced Therapy Medicinal Product for use in Humans
Source: Cells. 2021 Jul 27;10(8):1908. doi: 10.3390/cells10081908 (PMC8392403; doi:10.3390/cells10081908)
Supplement: Supplementary file 1 [file cells-10-01908-s001.zip › cells-1302136-supplementary.pdf]

Supplementary Materials

Supplementary Results

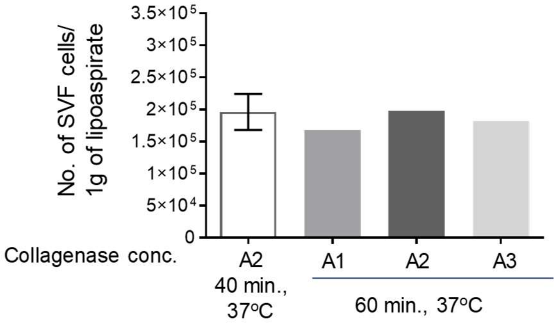

(a)

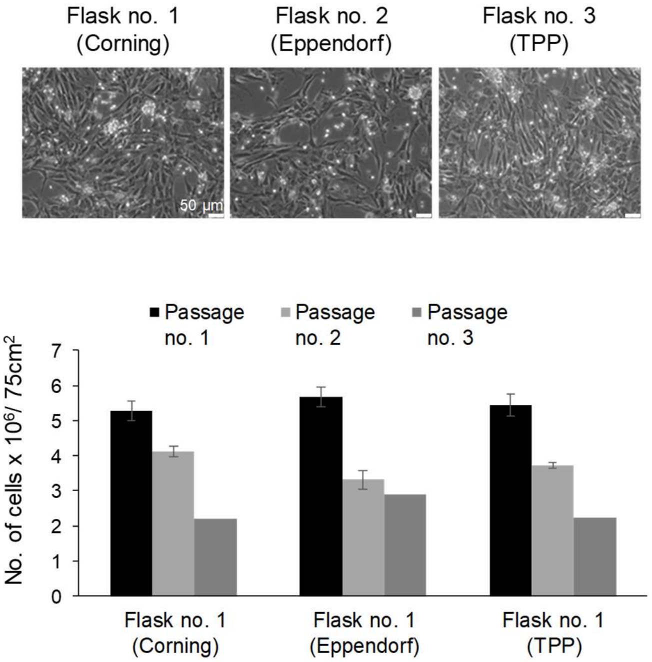

(b)

Figure S1. Optimization of isolation and culture of AT-MSCs. (a) Examination of digestion duration of AT. AT were incubated with A1, A2, A3 concentration of collagenase for 60 min at 37°C. The yield of isolation were presented as total number of SVF cells obtained per 1g of lipoaspirate and compared to yield of isolation obtained following incubation of AT with A2 concentration of collagenase for 40 min at 37°C. (b) Selection of culture flasks. Upper panel - Representative images of morphology of AT-MSCs (at passage no. 2) cultured on selected types of culture flasks produced by Corning, Eppendorf and TPP. Lower panel – Efficiency of AT-MSCs culture on selected types of culture flasks (Corning, Eppendorf and TPP) presented as number of cells detached from 75 cm^2 of growth area at passage no. 1, 2 and 3. Results are presented as mean±SD, N=3 (at passage 1 and 2) or N=1 (at pasaage 3).
